# Higher third-generation cephalosporin prescription proportion is associated with lower probability of reducing carbapenem use: a nationwide retrospective study

**DOI:** 10.1186/s13756-018-0302-8

**Published:** 2018-01-22

**Authors:** Allison Muller, Xavier Bertrand, Anne-Marie Rogues, Muriel Péfau, Serge Alfandari, Rémy Gauzit, Catherine Dumartin, Houssein Gbaguidi-Haore, on behalf of the ATB-RAISIN network steering committee, Serge Alfandari, Anne Berger-Carbonne, Xavier Bertrand, Sandrine Boussat, Anne-Claude Crémieux, Catherine Dumartin, Pascal Fascia, Rémy Gauzit, Marine Giard, Anne Ingels, François L’Hériteau, Ludivine Lacavé, Patricia Le Gonidec, Anaïs Machut, Sylvie Maugat, Lory Mouchot, Muriel Péfau, Anne-Marie Rogues, Benoît Schlemmer, Hélène Sénéchal, Sophie Touratier

**Affiliations:** 1 University Hospital of Besançon, Infection Control Department, F-25030 Besançon, France; 2 University Bourgogne-Franche-Comte, UMR 6249 Chrono-Environnement, F-25030 Besançon, France; 3 University Bordeaux, Inserm, Bordeaux Population Health Research Center, Team Pharmacoepidemiology, UMR 1219, F-33000 Bordeaux, France; 4 CHU Bordeaux, Southwestern Centre for Infection Prevention and Control, F-33000 Bordeaux, France; 5 Gustave Dron Hospital, Infectious Diseases Department, F-59208 Tourcoing, France; 6 AP-HP, Cochin University Hospital, Infectious Diseases Department, F-75014 Paris, France; 7 Service d’Hygiène Hospitalière, Centre Hospitalier Régional Universitaire, Hôpital Jean Minjoz, 3 Bd Fleming, 25030, Besançon, Cedex, France

**Keywords:** Carbapenem, Antibiotic use, Antibiotic stewardship, ESBL, Multidrug-resistant bacteria

## Abstract

**Background:**

The ongoing extended spectrum β-lactamase-producing *Enterobacteriaceae* (ESBL-PE) pandemic has led to an increasing carbapenem use, requiring release of guidelines for carbapenem usage in France in late 2010. We sought to determine factors associated with changes in carbapenem use in intensive care units (ICUs), medical and surgical wards between 2009 and 2013.

**Methods:**

This ward-level multicentre retrospective study was based on data from French antibiotic and multidrug-resistant bacteria surveillance networks in healthcare facilities. Antibiotic use was expressed in defined daily doses per 1000 patient-days. Factors associated with the reduction in carbapenem use (yes/no) over the study period were determined from random-effects logistic regression model (493 wards nested within 259 healthcare facilities): ward characteristics (type, size…), ward antibiotic use (initial antibiotic use [i.e., consumption of a given antibiotic in 2009], initial antibiotic prescribing profile [i.e., proportion of a given antibiotic in the overall antibiotic consumption in 2009] and reduction in the use of a given antibiotic between 2009 and 2013) and regional ESBL-PE incidence rate in acute care settings in 2011.

**Results:**

Over the study period, carbapenem consumption in ICUs (*n* = 85), medical (*n* = 227) and surgical wards (*n* = 181) was equal to 73.4, 6.2 and 5.4 defined daily doses per 1000 patient-days, respectively. Release of guidelines was followed by a significant decrease in carbapenem use within ICUs and medical wards, and a slowdown in use within surgical wards. The following factors were independently associated with a higher probability of reducing carbapenem use: location in Eastern France, higher initial carbapenem prescribing profile and reductions in consumption of fluoroquinolones, glycopeptides and piperacillin/tazobactam. In parallel, factors independently associated with a lower probability of reducing carbapenem use were ICUs, ward size increase, wards of cancer centres, higher initial third-generation cephalosporin (3GC) prescribing profile and location in high-risk regions for ESBL-PE.

**Conclusions:**

Our study suggests that a decrease in 3GCs in the overall antibiotic use and the continuation of reduction in fluoroquinolone use, could allow reducing carbapenem use, given the well-demonstrated role of 3GCs and fluoroquinolones in the occurrence of ESBL-PE. Thus, antibiotic stewardship programs should target wards with higher 3GC prescription proportions to reduce them.

## Background

The ongoing extended spectrum β-lactamase (ESBL)-producing *Enterobacteriaceae* (ESBL-PE) pandemic, mainly *Escherichia coli*, has led to an increasing use of carbapenems, which are considered as ‘last-line’ treatment against these multidrug-resistant (MDR) bacteria. This increasing carbapenem consumption worldwide [1–3], has accelerated the occurrence and spread of carbapenemase-producing *Enterobacteriaceae* (CPE) [4], which can lead to situations of therapeutic dead end. In France, the number of CPE cases has been increasing since 2010, and in an epidemic pattern in some regions [5]. In addition, more and more cases are no longer related to a foreign country [6]: from 29% of cases in 2010 to 58% in 2015. Lastly, according to the classification of the European survey of CPE (EuSCAPE), France is currently at epidemiological stage 4 of CPE spread [7], which corresponds to ‘multiple epidemiologically-related outbreaks occurring in different health districts, suggesting an interregional autochthonous inter-institutional transmission’. Saving antibiotic resources, particularly carbapenems, has become a global public health priority [8, 9], especially since the pipeline of new antibiotics is drying up [10]. Therefore, French guidelines for carbapenems have been released in late 2010 to limit their use and improve the prescribing practices of this antibiotic class [11]. In French healthcare facilities (HCFs), carbapenem consumption has undergone a significant rise of 145% between 2000 and 2013 [12]: from 0.014 to 0.033 defined daily doses (DDDs) per 1000 inhabitants and per day. This high consumption level is mainly observed in intensive care units (ICUs) [13], although carbapenem use according to hospital wards has been scarcely studied. In this study, we sought to evaluate changes in carbapenem use in ICUs, medical and surgical wards of French HCFs between 2009 and 2013, and to determine factors associated with the reduction in use of this antibiotic class.

## Methods

### Design, setting and study period

This multicentre retrospective study at the ward level was mainly based on data from the ATB-RAISIN network (RAISIN stands for network for warning, investigation and surveillance of nosocomial infections), which is responsible for the surveillance of antibiotic consumption in French HCFs. We included a stable cohort of ICUs, medical and surgical wards of HCFs that participated in the surveillance every year from 2009 to 2013 on a voluntary basis. The geographical location of these HCFs refers to Coordinating Centres for Nosocomial Infection Control, distributed in five areas (North, West, South-west, East and South-east), each including several administrative regions.

### Data collection

The ATB-RAISIN surveillance network retrospectively collected each year various types of data: (i) ward characteristics (type, size in number of beds, type of HCF from which it was derived, geographical location, and activity, expressed in number of patient-days), and (ii) inpatient antibiotic use, expressed in DDDs per 1000 patient-days (PD), in accordance to the Anatomical Therapeutic Chemical-DDD classification from the WHO [14]. Data on antibiotic use (dispensing data) were retrieved from pharmacy records, as described elsewhere [15]. Such data related to all antibiotic prescriptions whatever the context of treatment initiation (empirical or based on bacteriological results). Besides carbapenems (J01DH) and the overall antibiotic consumption (J01 + P01AB + J04AB02), the following antibiotics were studied: third-generation cephalosporins (3GCs, J01DD + J01DE + J01 DC07), fluoroquinolones (J01MA), aminoglycosides (J01G), aminopenicillins ± β-lactamase inhibitors (J01CA01 + 02 + 04 + J01CR01 + 02), imidazoles (J01XD + P01AB), glycopeptides (J01XA), piperacillin/tazobactam (J01CR05) and other potential alternatives to carbapenems for treating ESBL-PE infections (fosfomycin J01XX01, nitrofurantoin J01XE01, pivmecillinam J01CA08, temocillin J01CA17, tigecycline J01AA12 and cefoxitin J01 DC01) [16]. Moreover, the BMR-RAISIN network, which is responsible for the national surveillance of MDR bacteria in HCFs, provided data on ESBL-PE in acute care settings aggregated at the level of French regions. These data allowed taking into account the regional context in which a given ward operated in terms of ESBL-PE risk. Data generated from the laboratory, including antimicrobial susceptibility test results were only available for a few HCFs. Therefore, these data could not be considered in this work.

### Data analysis

Temporal trends in carbapenem use within wards were explored by calculating two indicators: the relative change (and its 95% confidence interval [CI]) and the annual growth rate of this consumption (obtained by dividing the relative change by the number of years of the relevant time period). These indicators were computed for various time periods: before the release of guidelines (2009–10), after the release of guidelines (2011–13) and over the whole of the study period (2009–13). These guidelines [11] aimed to specify indications, at the time, for the four available carbapenem molecules (doripenem has since been withdrawn from the French market) with the following rules of good clinical practice: carbapenem prescribing should be limited to nosocomial infections due to MDR Gram-negative bacteria, absence of a carbapenem-sparing alternative, empirical treatment with 48–72 h re-assessment and de-escalation using carbapenem-sparing regimen and limitation of ertapenem to ESBL-PE urinary tract infections treatment without alternative. We assumed that these guidelines, released in late 2010, were gradually implemented from 2011 within French HCFs through their antibiotic stewardship programs (ASPs) and driven by their ASP committee, especially the mandatory antibiotic advisor. To take into account the clustered structure of data (wards nested within HCFs), a random-effects logistic regression analysis was performed to determine factors associated with the reduction in carbapenem use. Indeed, traditional regression techniques, by ignoring this data structure and possible correlation of data, may lead to biased statistical inferences [17]. The binary outcome variable was the reduction in carbapenem use (yes/no) between 2009 and 2013. Independent variables were the following:

- ward characteristics;
- ward antibiotic use: initial antibiotic use (i.e., consumption of a given antibiotic in 2009), initial antibiotic prescribing profile (i.e., proportion in percent of a given antibiotic in the overall antibiotic consumption in 2009) and reduction in the use of a given antibiotic (binary variable: yes/no) between 2009 and 2013;
- regional ESBL-PE incidence rate in acute care settings in 2011, which was converted in binary data with the median as cut-off: a given ward was located in ‘high-risk region’ or in ‘low-risk region’ for ESBL-PE.

First, a univariable random-effects logistic regression analysis was carried out to assess crude relationship between the outcome variable and each independent variable. Second, to determine factors independently associated with the outcome, a multivariable random-effects logistic regression model was built by introducing all independent variables with a *p* value of less than 0.20 in univariable analysis except ‘type of ward’ and ‘initial overall antibiotic use’ that were forced into the model due to a strong presumption of confounding effect. A *p* value of less than 0.05 was considered as statistically significant. Analyses were conducted using the software package Stata, version 14.1 (Stata Corp., College Station, TX, USA).

## Results

### Ward characteristics

Data on participating wards are presented Table 1. A total of 493 wards from 259 HCFs (approximately 10% of French HCFs) were included in this study (85 ICUs, 227 medical wards and 181 surgical wards). These wards were from general hospitals at 78.8%, 61.2% and 52.5% for ICUs, medical and surgical wards, respectively.

**Table 1:** Characteristics of participating hospital wards across France

Characteristic	ICU wards, *N* = 85	Medical wards, *N* = 227	Surgical wards, *N* = 181
No. of beds in 2013, median (interquartile range)	12 (9–16)	72 (36–150)	63 (44–99)
Type of HCF			
- University hospital	4 (4.7%)	4 (1.8%)	4 (2.2%)
- General hospital	67 (78.8%)	139 (61.2%)	95 (52.5%)
- Private hospital	9 (10.6%)	51 (22.5%)	71 (39.2%)
- Cancer centre	0	6 (2.6%)	6 (3.3%)
- Local hospital	0	17 (7.5%)	0
- Rehabilitation and long-term care facility	0	5 (2.2%)	0
- Military hospital	5 (5.9%)	5 (2.2%)	5 (2.8%)
Geographical area			
- North	30 (35.3%)	50 (22%)	45 (24.9%)
- West	9 (10.6%)	25 (11%)	17 (9.4%)
- East	14 (16.5%)	45 (19.8%)	36 (19.9%)
- South-east	12 (14.1%)	33 (14.5%)	31 (17.1%)
- South-west	20 (23.5%)	74 (32.6%)	52 (28.7%)

*ICU* intensive care unit; *HCF* type of healthcare facility from which the hospital ward included in the study was derived

### Trends in antibiotic use

**Fig. 1:**
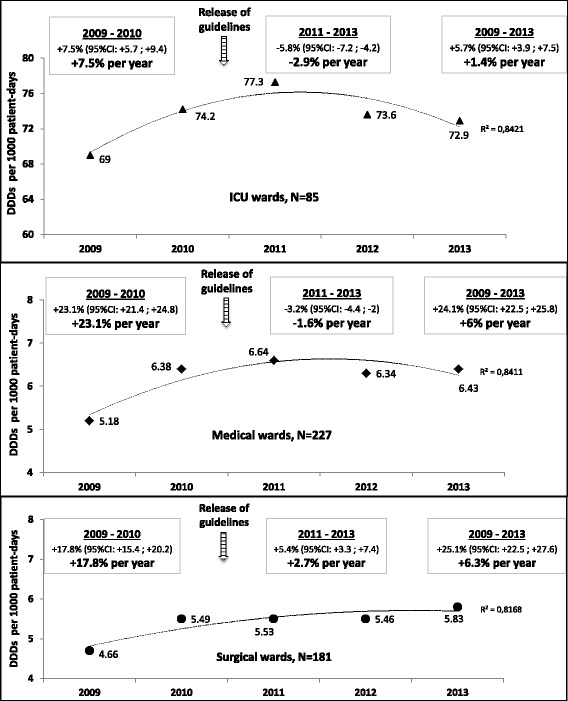
Changes in carbapenem use in hospital wards across France from 2009 to 2013. For each time period (2009–10, 2011–13 and 2009–13), the relative change and the annual growth rate of carbapenem consumption (in bold) were computed. *CI*, confidence interval; *DDD* defined daily dose

As the overall antibiotic consumption during the study period (2009–13), carbapenem consumption was higher in ICUs than in medical and surgical wards (Table 2): 73.4, 6.2 and 5.4 DDD/1000 PD, respectively. Additionally, in terms of initial carbapenem prescribing profile, data (medians) were the following: 3.8% (range, 0.2 to 14.6%) in ICUs, 0.5% (range, 0 to 6%) in medical wards and 0.4% (range, 0 to 4%) in surgical wards. Between 2009 and 2013, the overall antibiotic use slightly decreased in ICUs (from 1487 to 1479 DDD/1000 PD; − 0.52%) and in medical wards (from 622 to 616 DDD/1000 PD; − 0.89%), whereas it exhibited a moderate increase in surgical wards (from 592 to 625 DDD/1000 PD; + 5.68%). Regarding carbapenem use over the study period, relative changes showed an increase across the three types of wards (Fig. 1): ICUs (+ 5.65%), medical wards (+ 24.1%) and surgical wards (+ 25.1%). Interestingly, this increase did not affect 201 out of 493 (40.8%) wards included, in which we observed a reduction in carbapenem use: 38.8% (33/85) of ICUs, 41.9% (95/227) of medical wards and 40.3% (73/181) of surgical wards. Exploring temporal trends in carbapenem use before the release of guidelines (2009–10) revealed a statistically significant increase across the three types of wards (Fig. 1): ICUs (+ 7.5%), medical wards (+ 23.1%) and surgical wards (+ 17.8%). The time period after the release of guidelines (2011–13) was followed by, on the one hand, a trend reversal in carbapenem use in ICUs and medical wards (negative annual growth rate of consumption: − 2.9% and − 1.6%, respectively) and, on the other hand, a slowdown in this consumption in surgical wards (annual growth rate of consumption declined from + 17.8% to + 2.7%). We should also point out that the statistically significant decrease in carbapenem use after the release of guidelines within ICUs (− 4.9% [95% CI, − 6.4 to − 3.3]) and medical wards (− 4.6% [95% CI, − 5.8 to − 3.4]) was more marked in 2012. By contrast, the carbapenem use within surgical wards only displayed a downward trend (− 1.3% [95% CI, − 3.2 to + 0.7]) between 2011 and 2012, which did not reach statistical significance. As regards other antibiotic use (Table 2), in almost all cases where an upward trend was noted over the five-year study period, this increase was greater in surgical wards than in ICUs and medical wards (e.g., 3GC use). Conversely, in case of downward trend recorded, the decrease was absent or lesser in surgical wards than in other wards (e.g., fluoroquinolone use). It should also noteworthy that initial 3GC prescribing profile medians varied as follows: 13.3% (range, 6.2 to 28.2%) in ICUs, 8.7% (range, 1.4 to 30.4%) in medical wards and 4.8% (range, 0 to 19.3%) in surgical wards. Moreover, initial fluoroquinolone prescribing profile medians were: 13.7% (range, 2.6 to 33.3%) in ICUs, 16.6% (range, 5.7 to 32.5%) in medical wards and 13.1% (range, 2.8 to 33.4%) in surgical wards.

**Table 2:** Use of carbapenems and other antibiotic classes in hospital wards across France, 2009–2013

Antibiotic use, DDDs per 1000 patient-days (RC)^a^	ICU wards, *N* = 85	Medical wards, *N* = 227	Surgical wards, *N* = 181
All antibiotics	1487 (−0.52%)	615 (−0.89%)	607 (+ 5.68%)
Carbapenems	73.4 (+ 5.65%)	6.2 (+ 24.1%)	5.4 (+ 25.1%)
- Imipenem	62.1 (−0.74%)	5.3 (+ 13.9%)	4.2 (+ 27.5%)
- Meropenem	5.3 (+ 285%)	0.36 (+ 195%)	0.23 (+ 109%)
- Ertapenem	4.87 (−20.4%)	0.52 (+ 45.3%)	0.86 (− 13.3%)
- Doripenem	1.17 (−16.7%)	0.04 (+ 208%)	0.06 (+ 1125%)
Aminopenicillins ± β-lactamase inhibitors	417 (−2.86%)	318 (−1.65%)	268 (+ 0.94%)
Piperacillin/tazobactam	90.1 (+ 34.7%)	8.6 (+ 79%)	8 (+ 109%)
Third-generation cephalosporins	205.9 (+ 0.78%)	60.4 (+ 18.9%)	38.1 (+33.2%)
Fluoroquinolones	177.2 (−24.2%)	88.4 (− 21.3%)	75.1 (−13.1%)
Aminoglycosides	106.1 (+3.35%)	12.4 (−6.98%)	25.4 (+ 13.8%)
Glycopeptides	59.6 (−23.2%)	9.8 (−5.1%)	10.3 (+ 23.6%)
Imidazoles	61.9 (−3.93%)	20.2 (+ 20.6%)	39.6 (+ 9.96%)

*DDD* defined daily dose; *ICU* intensive care unit; *RC* relative change (%) between 2009 and 2013

^a^Pooled means in DDDs per 1000 patient-days and relative changes in percent

### Factors associated with the reduction in carbapenem use

Statistical analysis results are reported in Table 3. Surprisingly, univariable analysis detected no significant difference in reducing carbapenem use according to ward characteristics. In contrast, as regards antibiotic use, wards that recorded a decline in their overall antibiotic use reduced more often their carbapenem use (univariable analysis, *p* = 0.003). In multivariable analysis (Table 3), the following factors were independently associated with a higher probability of reducing carbapenem use: a geographical location in Eastern France in comparison with South-eastern area of the country, a higher initial carbapenem prescribing profile and the reductions in consumption of fluoroquinolones, glycopeptides and piperacillin/tazobactam. Furthermore, other factors were independently associated with a lower probability of reducing carbapenem use: ICU wards (vs. medical wards), ward size increase, wards of cancer centres (vs. wards of general hospitals), a higher initial 3GC prescribing profile and location in a high-risk region for ESBL-PE.

**Table 3:** Factors associated with the reduction in carbapenem use from random-effects logistic regression model

Variable	Univariable analysis		Multivariable analysis		
	OR	*p*	OR	*p* ^*a*^	95% CI
Type of ward (reference: Medical)					
ICU	0.94	0.83	***0.07***	***0.005***	***0.01–0.46***
Surgical	0.95	0.81	0.66	0.19	0.35–1.23
Ward size in 2013, per increase of 50 beds	0.90	0.14	***0.79***	***0.02***	***0.64–0.97***
Type of HCF (reference: General hospital)					
University hospital	0.42	0.35	0.28	0.25	0.03–2.39
Private hospital	1.06	0.85	0.66	0.28	0.31–1.40
Cancer centre	0.37	0.29	***0.09***	***0.04***	***0.01–0.88***
Local hospital	2.33	0.18	1.19	0.83	0.25–5.54
Rehabilitation and long-term care facility	0.38	0.50	0.24	0.40	0.01–6.59
Military hospital	0.43	0.34	0.18	0.09	0.02–1.28
Geographical area (reference: South-east)					
North	1.42	0.42	2.33	0.09	0.88–6.16
West	1.15	0.80	1.09	0.90	0.30–3.94
East	1.93	0.15	***3.11***	***0.03***	***1.10–8.77***
South-west	1.78	0.17	1.57	0.36	0.60–4.12
Antibiotic use, DDDs per 1000 patients-days					
Initial CBP use^b^	1.01	0.01	0.97	0.08	0.95–1.00
Initial prescribing profile of CBPs^c^	1.22	0.002	***3.16***	***< 0.001***	***1.88–5.34***
Initial 3GC use^b^	1.00	0.79			
Initial prescribing profile of 3GCs^c^	0.97	0.16	***0.89***	***0.002***	***0.83–0.96***
Reduction in 3GC use^d^	1.34	0.23			
Initial FQ use^b^	1.00	0.25			
Initial prescribing profile of FQs^c^	1.02	0.29			
Reduction in FQ use^d^	2.21	0.004	***2.3***	***0.02***	***1.17–4.53***
Initial AG use^b^	1.00	0.50			
Initial prescribing profile of AGs^c^	0.97	0.44			
Reduction in AG use^d^	1.56	0.06	1.13	0.67	0.65–1.96
Initial AP ± BLI use^b^	1.00	0.83			
Initial prescribing profile of AP±BLIs^c^	1.00	0.99			
Reduction in AP ± BLI use^d^	1.10	0.68			
Initial Imidazole use^b^	1.00	0.98			
Initial prescribing profile of imidazoles^c^	0.97	0.45			
Reduction in imidazole use^d^	1.65	0.03	1.50	0.15	0.87–2.59
Initial GP use^b^	1.00	0.95			
Initial prescribing profile of GPs^c^	0.97	0.61			
Reduction in GP use^d^	1.83	0.01	***1.98***	***0.02***	***1.13–3.45***
Initial TZP use^b^	1.00	0.99			
Initial prescribing profile of TZP^c^	0.95	0.33			
Reduction in TZP use^d^	4.24	< 0.001	***4.02***	***< 0.001***	***1.99–8.14***
Initial use of other alternatives to CBPs^b^	1.00	0.88			
Initial prescribing profile of other alternatives to CBPs^c^	1.31	0.25			
Reduction in the use of other alternatives to CBPs^d^	1.07	0.79			
Initial overall antibiotic use^b^	1.00	0.72	1.00	0.97	0.99–1.01
Reduction in the overall antibiotic use^d^	2.02	0.003	1.38	0.29	0.76–2.51
Region with high ESBL-PE incidence rate in acute care settings^e^	0.65	0.12	***0.40***	***0.01***	***0.19–0.82***

*OR* odds ratio, *CI* confidence interval, *ICU* intensive care unit, *HCF* healthcare facility, *DDD* defined daily dose, *CBP* carbapenem, *3GC* third-generation cephalosporin, *FQ* fluoroquinolone, *AG* aminoglycoside, *AP ± BLI* aminopenicillin ± β-lactamase inhibitor, *GP* glycopeptide, *TZP* piperacillin/tazobactam. *ESBL-PE* extended-spectrum β-lactamase-producing *Enterobacteriaceae*

^a^Significant associations (*p* < 0.05) from multivariable analysis are highlighted in bold italic

^b^Consumption of a given antibiotic in 2009

^c^Proportion (%) of a given antibiotic in the total antibiotic consumption in 2009

^d^Reduction in the consumption of a given antibiotic between 2009 and 2013

^e^Data from the French multidrug-resistant bacteria surveillance network; BMR-RAISIN, 2011

## Discussion

### Antibiotic use and potential impact of guidelines on carbapenem use

In comparison with the meta-analysis of Bitterman et al. [18], our study showed that antibiotic consumption in ICUs and medical wards was lower in France than in other countries worldwide: for overall antibiotic use (1487 vs. 1563 DDD/1000 PD in ICUs and 615 vs. 677 DDD/1000 patient-days in medical wards), as well as for specific antibiotic classes, including carbapenems (73.4 vs. 109 DDD/1000 PD in ICUs and 6.2 vs. 16 DDD/1000 PD in medical wards). In the current study, the release of guidelines for carbapenems in late 2010 was followed by a significant decrease or a slowdown in their usage in French hospital wards. This finding suggests a potential impact of these guidelines on reducing carbapenem use. Additionally, the magnitude of this impact appears more marked in ICUs and medical wards than in surgical wards. These guidelines fitted into the framework of the French national antibiotic action plans (initiated in 2001, the latest iteration being 2011–2016) [9] from the French Ministry of Health. The latter required from each HCF: (i) implementation of an ASP since 2002 and (ii) an annual public reporting of the compliance since 2007 with specific antimicrobial stewardship performance indicators, in order to enhance rational antibiotic use [19]. As with carbapenems, trends in the usage of other antibiotics suggest that efforts for curbing antibiotic overuse are more frequently successful in ICUs and medical wards than in surgical wards. This finding is consistent with a study carried out at the Zurich University Hospital, which reported a higher proportion of inappropriate therapeutic antibiotic use in surgical wards (49.3%), in comparison with ICU (32.4%) and medical wards (32.9%) [20]. Other studies [21, 22] showed in surgeons a poor adherence to ASP, sometimes even following an educational intervention program. Some factors specific to surgeons may explain this fact, such as lack of extensive training in infectious diseases and antibiotic usage of most surgical training programs [23]. Therefore, a special effort should be made for surgeons in ASP interventions that aim at reducing carbapenem use.

### Factors modifying the probability of reducing ward-level carbapenem use

The multivariable analysis identified five factors positively associated with the reduction in carbapenem use. First, a geographical location in Eastern France was associated with a higher probability of reducing carbapenem use compared with a location in South-eastern of the country. This could be a result of specific local situations. We could cite as an example, the increasing spread of CPE, in a region previously hit by a major vancomycin-resistant enterococci outbreak [24], which may have prompted a better compliance with prudent carbapenem use to lower selection pressure. All other things being equal, including initial overall antibiotic use, a 1% increase in initial carbapenem prescribing profile was associated with more than threefold increase in the probability of reducing carbapenem use. Such an observation could be due to the fact that it is probably easier to reduce carbapenem use within wards with high proportions of carbapenems in the initial overall antibiotic use than within wards that use them sparingly. Indeed, in these wards with a high initial carbapenem prescribing profile, there was likely a greater room for improvement in terms of proper carbapenem use, notably through de-escalation strategies, which are still insufficiently implemented [25]. Lastly, wards, which reduced their consumptions of fluoroquinolones, glycopeptides and piperacillin/tazobactam, reduced more frequently their carbapenem use. Maybe this reflects what is covered by the positive and significant association between the reduction in overall antibiotic use and the reduction in carbapenem use in univariable analysis. This association was not significant in multivariable analysis. Furthermore, about the variable ‘reduction in fluoroquinolone use’, this could be also interpreted as a result of the involvement of fluoroquinolones in the occurrence of antibiotic-resistant bacteria [26], including ESBL-PE. Indeed, the reduction in fluoroquinolone use decreased the ESBL-PE risk and therefore the recourse to carbapenems. Moreover, this reduction in fluoroquinolone use is probably due to multiple ASP interventions within French HCFs and release of national guidelines, especially for urinary tract infections [27–29]. Clearly, the decrease in fluoroquinolone use over the study period has been partly achieved thanks to a shift of the antibiotic prescribing towards 3GCs and piperacillin/tazobactam, of which consumptions increased while overall antibiotic use remained almost stable. Besides, the positive association between the reduction in piperacillin/tazobactam use and the reduction in carbapenem use was surprising in view of the potential role of piperacillin/tazobactam as a carbapenem-sparing antibiotic. An optimistic explanation could be the ‘virtous cycle’ hypothesis, with a global improvement in prescribing, resulting in a decrease of all previously overprescribed antibiotics. In parallel, five other factors were negatively associated with reduction in carbapenem use. ICUs and wards of cancer centres had probabilities of reducing carbapenem use which were about 90% lower than those of medical wards and wards of general hospitals, respectively. Such a finding is consistent, given a higher severity and a more frequent previous antibiotic exposure in patients in these acute care settings, leading to a greater risk for developing severe infections with resistant Gram-negative bacteria [30]. Therefore, carbapenem prescribing can be less frequently avoidable. A 50-bed increase in ward size was associated with a 21% decrease in the probability of reducing carbapenem use. To explain this result, we assume that the number of doctors prescribing antibiotics increases with the ward size. It is likely that interventions conducted by ASP committees, which aim at promoting the proper use of carbapenems are harder to implement within larger wards, especially since the heterogeneity of antibiotic prescribing is possibly higher [31]. Lastly, a 1% increase in the initial prescribing profile of 3GCs was associated with 11% decrease in the probability of reducing carbapenem use, and this for the same level of initial overall antibiotic use. Likewise, wards located in high-risk regions for ESBL-PE exhibited a 60% decrease in the probability of reducing carbapenem use. The explanation for these two last findings is to be found in the ESBL-PE risk, which is promoted by 3GC use [26], and thus leads to more frequent carbapenem use. It should be recalled that 3GC use remained stable or increased between 2009 and 2013 across the three types of wards. Hence, the reduction in 3GC use appears as an essential factor in future ASPs aiming to reduce carbapenem prescribing. To a lesser extent, this is also the case for the reduction in fluoroquinolone use, which is already in progress and must be continued. For example, the retrospective multicentre study from Batard et al. [32], regarding emergency department patients treated for community-acquired pneumonia, revealed that use of 3GCs and fluoroquinolones was avoidable in 70% and 52% of cases, respectively. These antibiotics could be replaced with aminopenicillins ± β-lactamase inhibitors. Furthermore, recent applications of matrix-assisted laser desorption ionization–time of flight (MALDI-TOF) mass spectrometry (MS) for rapid detection of antibiotic resistance offer promising perspectives for optimizing antibiotic prescribing in clinical practice [33, 34]. In other terms, MALDI-TOF MS, in addition to being a technology for pathogen identification, could be a valuable tool for early therapeutic guidance by allowing for example an early de-escalation whenever possible. Nevertheless, in all cases, the issue of adherence to the guidelines is central to the success of such developments.

### Limitations

This ward-level multicentre retrospective study is subject to various limitations. First, no patient-level confounding factor has been taken into account. Consequently, case-mix and potential ecological bias inherent to this type of study could not be controlled. Second, associations are suggestive and do not necessarily imply a cause-effect relationship. Therefore, these findings must be confirmed through randomized controlled intervention studies exploring joint effects of individual- and group-level factors. Third, we had no data regarding ASPs in HCFs enrolled in the study. As a result, we could not measure the implementation of carbapenem use guidelines. Fourth, DDD measurement presents biases mentioned elsewhere [35], including as a metric to study the impact of antibiotic stewardship. Fifth, no data was available regarding compliance with infection prevention and control measures. Indeed, hand hygiene, barrier precautions and carrier screening, by avoiding the spread of MDR bacteria, may influence carbapenem use. Additionally, none information on the occurrence of MDR bacteria outbreaks within HCFs was available in this study.

## Conclusions

Despite these caveats, our study, carried out on a national scale, in a large number of hospital wards, suggests that a decrease in 3GC proportion in the overall antibiotic use, as well as the continuation of the reduction in fluoroquinolone use, could allow reducing ward-level carbapenem use. Thus, in their future interventions, ASPs should target not only wards with a higher carbapenem use, but also those with a higher 3GC prescription proportion to conduct actions focusing on both 3GCs and carbapenems to preserve the effectiveness of these antibiotics in French HCFs.

## Abbreviations

3GC: Third-generation cephalosporin
95% CI: Confidence interval
ASP: Antibiotic stewardship program
CPE: Carbapenemase-producing *Enterobacteriaceae*
DDD: Defined daily dose
ESBL-PE: Extended spectrum β-lactamase-producing *Enterobacteriaceae*
HCF: Healthcare facility
ICU: Intensive care unit
MALDI-TOF MS: Matrix-assisted laser desorption ionization–time of flight mass spectrometry
MDR: Multidrug-resistant
PD: Patient-day
